# Pharmacokinetics of solifenacin in pediatric populations with overactive bladder or neurogenic detrusor overactivity

**DOI:** 10.1002/prp2.684

**Published:** 2020-11-24

**Authors:** Stacey Tannenbaum, Martin den Adel, Walter Krauwinkel, John Meijer, Adriana Hollestein‐Havelaar, Frank Verheggen, Donald Newgreen

**Affiliations:** ^1^ Astellas Pharma Global Development Inc. Northbrook IL USA; ^2^ Astellas Pharma Europe B.V. Leiden The Netherlands

**Keywords:** nephrology – urology, pediatrics – children, pharmacokinetics

## Abstract

The aim of this investigation was to characterize and compare the pharmacokinetics (PK) of the antimuscarinic drug solifenacin in pediatric patients with overactive bladder (OAB) or neurogenic detrusor overactivity (NDO) utilizing data from three phase III trials. LION was a placebo‐controlled, 12‐week trial in children (5–<12 years) and adolescents (12–<18 years) with OAB. MONKEY and MARMOSET were open‐label, 52‐week trials in children and adolescents or younger children (6 months–<5 years), respectively, with NDO. During the trials, solifenacin doses could be titrated to weight‐adjusted pediatric equivalent doses (PEDs) of 2.5, 5, 7.5, or 10 mg day^–1^. Nonlinear mixed effects modeling was used to develop population PK models to characterize the PK in patients with either OAB or NDO. Overall, 194 children and adolescents received solifenacin. At the time of PK sampling, the majority (119/164 [72.6%] patients) were receiving PED10 once daily. All population models included first‐order oral absorption, a lag time, and interindividual variability. PK analysis showed that apparent clearance was similar in both patient populations. Mean apparent oral plasma clearance (CL/F), apparent volume of distribution during the terminal phase (V_z_/F), and terminal half‐life (t_1/2_) were higher in adolescents than in children, but median time to maximum plasma concentration (t_max_) was similar. Dose‐normalized exposure results were similar for both younger and older patients with OAB or NDO. In conclusion, population PK modeling was used to successfully characterize solifenacin PK in pediatric patients with OAB or NDO. Similar solifenacin PK characteristics were observed in both populations.

**Abbreviations**
ALAG: absorption lag time
AUC/D: dose‐normalized area under the plasma concentration‐time curve
C_max_/D: dose‐normalized maximum plasma concentration
C_trough_/D: dose‐normalized trough plasma concentration
CV: coefficient of variation
F1: relative bioavailability
FFM: Fat‐free mass
MVV: mean volume voided
NDO: Neurogenic detrusor overactivity
OAB: Overactive bladder
PK: pharmacokinetics
Q/F: apparent intercompartmental clearance
SD: standard deviation
SE: standard error
V3/F: apparent peripheral volume of distribution

## 1 INTRODUCTION

Overactive bladder (OAB) syndrome is defined in children and adolescents, as in adults, on the basis of symptoms of urinary urgency, with or without urgency urinary incontinence, usually with frequency and nocturia, if there is no proven infection or other obvious pathology.^1^, ^2^ Epidemiological studies have indicated that OAB is a highly prevalent syndrome and between 3.2% and 16.6% of pediatric patients can be affected by OAB symptoms.^3^, ^4^, ^5^ Pediatric patients predominately experience the stressful symptoms of increased daytime frequency and urgency urinary incontinence.^6^

Neurogenic detrusor overactivity (NDO) in pediatric patients, as in adults, is defined as detrusor overactivity, characterized by involuntary detrusor contractions during the filling phase, which has a relevant neurological cause.^1^, ^7^ Patients can experience high intravesical pressures and/or vesicoureteral reflux, which can lead to irreversible renal damage, especially when accompanied by urinary tract infections and episodes of acute pyelonephritis.^8^

Some antimuscarinics have been recommended as first‐line pharmaceutical agents for treating adult patients with OAB symptoms or NDO.^9^, ^10^ However, only a few antimuscarinic medications are available for treating pediatric patients with either of these conditions.^11^, ^12^ For example, oxybutynin is approved within the EU for treating children who are > 5 years old, whereas trospium is only indicated in patients aged 12 years or older.^13^, ^14^

Several clinical investigations have demonstrated the efficacy and safety of the antimuscarinic, solifenacin, for treating adult patients with OAB and NDO.^15^, ^16^, ^17^, ^18^, ^19^ These positive findings have led to the approval of solifenacin for treating OAB symptoms in several regions worldwide, including Europe, the United States, and Japan (2.5, 5, and 10 mg tablets and 1 mg ml^–1^ oral suspension).^20^, ^21^, ^22^, ^23^ Further studies have also been conducted with a solifenacin suspension that has been formulated for use in pediatric patients. Solifenacin treatment led to statistically significant improvements in mean voiding frequency and mean volume voided (MVV) following administration to 34 children with newly diagnosed OAB and 138 children with therapy‐resistant OAB, respectively.^24^, ^25^ In addition, the results of phase III trials in children and adolescents with OAB showed that oral solifenacin suspension was superior to placebo in terms of MVV and was well tolerated over 52 weeks of treatment, with the majority of adverse events being mild or moderate in severity and no treatment‐related serious adverse events being reported.^26^, ^27^ No unexpected safety concerns were apparent when solifenacin suspension was administered to children and adolescents with NDO and the drug appeared to be an efficacious and well‐tolerated treatment, with a minority of patients reporting adverse events, when it was used to treat children with oxybutynin‐ or tolterodine‐refractory NDO.^28^, ^29^ Furthermore, two phase III open‐label trials in 99 pediatric patients with NDO showed that the use of solifenacin significantly increased maximum cystometric capacity and the antimuscarinic was well tolerated, with most adverse events being mild or moderate in severity.^30^ The results of these trials contributed to the European and US approval of oral solifenacin suspension for the treatment of pediatric patients (2–<18 years) with NDO.^31^, ^32^

Ascertaining the pharmacokinetics (PK) of potential drugs provides vital information that can be used to determine the optimal dosing regimen to be included in the drug label that informs prescribers. PK studies in healthy adults have shown that solifenacin is suitable for once‐daily administration,^33^ demonstrates high oral bioavailability,^34^ and exhibits PK properties that are not affected by food ingestion.^35^ In addition, solifenacin is mainly metabolized by hepatic cytochrome P450 3A4 (CYP3A4), predominately eliminated in the urine (approximately 70%, mostly as metabolites), and displays a high degree of plasma protein binding (approximately 98%, primarily to α_1_‐acid glycoprotein [AGP]).^36^

Anatomical, physiological, and biochemical differences between children and adults can have a profound effect on the PK and pharmacodynamics of a medication.^37^ Therefore, for any medication that will be used in pediatric patients, it is necessary to establish the PK of the drug in this specific population. Two single‐dose trials evaluated the PK of solifenacin in pediatric patients with OAB or NDO;^28^, ^38^ these trials indicated that similar solifenacin exposures are observed in both patient populations following the administration of a single weight‐adjusted dose of the drug.^28^

Three subsequent phase III clinical trials, which included PK assessments, have investigated the administration of multiple doses of oral solifenacin succinate suspension to pediatric patients with OAB or NDO. The efficacy and safety results from these trials are briefly mentioned above and have been previously reported.^26^, ^30^ The objective of the present investigation was to utilize the data from these trials to characterize the PK of solifenacin through the use of nonlinear mixed effects modeling.

## 2 MATERIALS AND METHODS

### 2.1 Ethics

Prior to any trial‐related screening procedures being performed, written informed consent was obtained from the parents and/or legal guardians of the patients in all three trials. Assent was obtained from the patients themselves where appropriate. The ethical, scientific, and medical appropriateness of the trials were reviewed by Independent Ethics Committees before they commenced. All three trials were performed in compliance with Good Clinical Practice.

### 2.2 Trial designs

**FIGURE 1:**
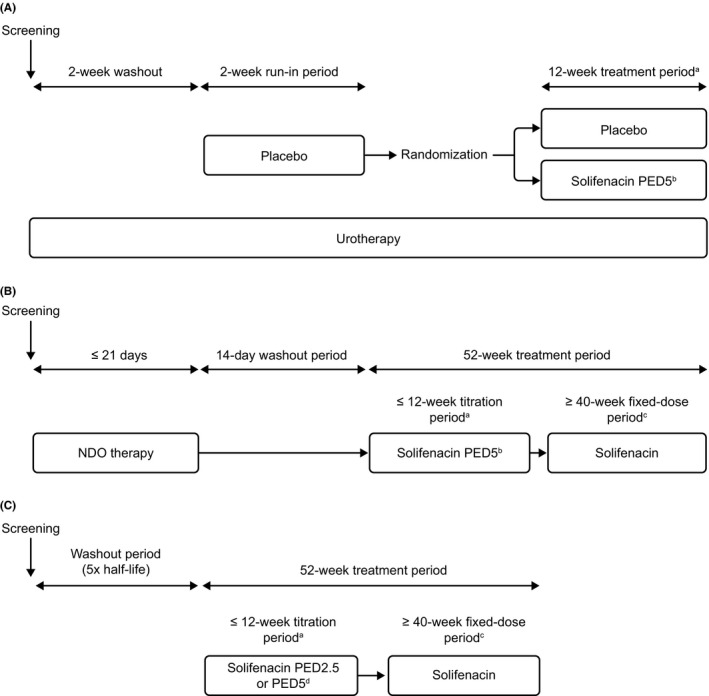
Trial designs for the LION (OAB population; (A), MONKEY (NDO population; (B), and MARMOSET (NDO population; (C) trials. NDO, neurogenic detrusor overactivity; OAB, overactive bladder; PED, pediatric equivalent dose. Figure 1A reprinted from Eur Urol, 71, Newgreen D, Bosman B, Hollestein‐Havelaar A, Dahler E, Besuyen R, Sawyer W, *et al*. Solifenacin in children and adolescents with overactive bladder: results of a phase 3 randomised clinical trial, 483‐90, Copyright (2017), with permission from Elsevier.^26^ Figure 1B and Cadapted from Franco, *et al*.^30^ ^a^Every 3 weeks, dose modification of trial drug could be performed to obtain doses of PED2.5, PED5, PED7.5, or PED10. ^b^Weight‐adjusted starting dose was equivalent to 5 mg in adults. ^c^The fixed‐dose assessment period started when the optimal dose for the patient was reached and it ended at week 52. ^d^Weight‐adjusted starting dose was equivalent to 2.5 or 5 mg in adults depending on patient age

The designs for all three trials are presented in Figure 1 and inclusion and exclusion criteria are presented in Table S1.

#### 2.2.1 The LION trial (ClinicalTrials.gov: NCT01565707)

This was a double‐blind, randomized, placebo‐controlled, sequential dose‐titration trial conducted in children (5–<12 years) and adolescents (12–<18 years) with OAB (Study 905‐CL‐076). The methodology has been presented elsewhere.^26^ Briefly, patients started urotherapy^39^ 4 weeks prior to randomization following completion of the screening procedures. After 2 weeks, a single‐blind 2‐week placebo run‐in period was started in combination with the ongoing urotherapy. After this run‐in period, eligible patients, who had not gained sufficient benefit from urotherapy alone, were randomized to receive 12 weeks of daily double‐blind treatment with either oral solifenacin succinate suspension or placebo. Patients initially received a weight‐adjusted once‐daily dose of solifenacin that was intended to give a similar exposure as steady‐state dosing of 5 mg to adults (pediatric equivalent dose [PED5]). Doses could be subsequently up‐ or down‐titrated every 3 weeks for up to 9 weeks, to enable the patients to receive their optimal weight‐adjusted dose of PED2.5, PED5, PED7.5, or PED10. Dose titrations were dependent on the efficacy (whether the patient was dry or not) and safety (whether the patient experienced a bothersome event that was possibly related to the trial drug) of the dose. Blood samples were collected for PK assessment at week 12 within 3 hours prior to dosing and 1‐3, 4‐5, and 7‐10 hours, and 2‐3 days after the last dose of trial drug. One additional blood sample was taken at week 12 to analyze the AGP level.

#### 2.2.2 The MONKEY trial (ClinicalTrials.gov: NCT01565694)

This was an open‐label, baseline‐controlled, sequential dose‐titration trial in children (5–<12 years) and adolescents (12–<18 years) with NDO (Study 905‐CL‐047). The methodology has been presented elsewhere.^30^ Briefly, following screening, patients continued to take their current NDO medication (alfuzosin, oxybutynin, propiverine, solifenacin, or tolterodine) for up to 21 days. After a subsequent 14‐day washout period, patients commenced treatment with daily doses of oral solifenacin succinate suspension. Patients initially received a weight‐adjusted once‐daily dose of PED5. Doses could be subsequently up‐ or down‐titrated every 3 weeks for up to 12 weeks, to enable the patients to receive their optimal weight‐adjusted dose of PED2.5, PED5, PED7.5, or PED10. Dose titrations were dependent on the efficacy (whether the patient was dry or not) and safety (whether the patient experienced intolerable adverse events) of the dose. The fixed‐dose assessment period started when the optimal dose for the patient was reached and it ended at week 52. PK sampling was performed at one visit or was spread across two visits that occurred on weeks 12, 24, and/or 36 (week 12 samples were only acquired if final dose titration occurred prior to week 12). The PK samples were taken within 3 hours prior to dosing and 1‐3, 4‐6, and 7‐10 hours post dose (four samples in total). One additional blood sample was taken at week 12, 24, or 36 to analyze the AGP level.

#### 2.2.3 The MARMOSET trial (ClinicalTrials.gov: NCT01981954)

This was an open‐label, baseline‐controlled, sequential dose‐titration trial in younger children (6 months–<5 years) with NDO (Study 905‐CL‐074). The methodology has been presented elsewhere.^30^ Briefly, patients who were receiving treatment with antimuscarinic agents and/or other prohibited medications required a washout period (equal to five times the medication half‐life) between screening and the start of trial drug. Subsequently, patients were treated with daily doses of oral solifenacin succinate suspension. Patients initially received a weight‐adjusted once‐daily dose of PED2.5 for children < 2 years or PED5 for children > 2 years. Doses could be subsequently up‐ or down‐titrated every 3 weeks for up to 12 weeks, to enable the patients to receive their optimal weight‐adjusted dose of PED2.5, PED5, PED7.5, or PED10. Dose titrations were dependent on the efficacy (whether the investigator considered that efficacy could be improved) and safety (whether the patient experienced bothersome adverse reactions) of the dose. The fixed‐dose assessment period started when the optimal dose for the patient was reached and it ended at week 52. PK sampling was performed at one visit or was spread across up to three visits (weeks 12, 24, and/or 36). The PK samples were taken within 3 hours prior to dosing and 1‐3, 4‐5, and 7‐10 hours post dose (four samples in total). One additional blood sample was taken at week 24 to analyze the AGP level.

### 2.3 Bioanalytical methods

For the PK assessments, samples of venous blood (2 mL, except 1 mL for the MARMOSET trial) were collected at the appropriate time points. The bioanalysis of solifenacin free base in heparin plasma was subsequently conducted using a validated liquid chromatography‐tandem mass spectrometry method. For the pretreatment, samples were extracted from plasma using solid‐supported liquid extraction involving a 200 mg 96‐well SLE + plate (Biotage, Uppsala, Sweden) and eluted using two aliquots of 0.8 mL methyl *tert*‐Butyl ether/dichloromethane (50:50 v/v). Solifenacin was subsequently separated from plasma constituents using ultra‐performance liquid chromatography (Waters Acquity, Milford, MA, USA) with a Phenomenex (Torrance, CA, USA) Kinetex pentafluorophenyl (PFP) column (100 Å; 50 x 2.1 mm; dp = 2.6 µm) coupled with an AB Sciex (Framingham, MA, USA) API 4000 mass spectrometer in the electrospray positive ion mode. For all of the trials, the precision (% coefficient of variation) of the assay was ≤ 7.4%, the accuracy (% relative error) varied between –4.9% and +4.5%, and the assay range was 0.2‐200 ng mL^–1^.

Analyses of serum AGP levels were performed by a central laboratory using a standardized immunoturbidimetric assay on a Roche Cobas C platform (Roche Diagnostics GmbH, Mannheim, Germany). For all trials, the precision of the assay was < 2.1%, accuracy was < 9.5%, and the assay range was 20‐600 mg dL^–1^.

### 2.4 PK and statistical analyses

Population PK modeling was performed using nonlinear mixed effects modeling software (NONMEM, version 7.3) and solifenacin PK parameters were summarized using descriptive statistics.

To support model stability for the LION trial, the PK data from this trial were pooled with the results from a single‐ascending dose trial (GIRAFFE; Study 905‐CL‐075; ClinicalTrials.gov: NCT01262391^38^), which involved frequent PK sampling and the same patient population (children and adolescents with OAB between the ages of 5 and < 18 years). The 42 patients enrolled in GIRAFFE received a single dose of solifenacin suspension that was three times higher than the calculated weight‐adjusted PED (PED2.5, PED5, or PED10). This dosing reflected the observation in adults that plasma concentrations of solifenacin increase approximately threefold under steady‐state conditions compared with a single dose; this dosing regimen was therefore adopted to obtain steady‐state plasma concentrations during this single‐dose trial.

## 3 RESULTS

Patient demographics and baseline characteristics from all three trials are summarized in Table 1. In total, 194 children and adolescents received treatment with solifenacin in the three trials.

**TABLE 1:** Patient demographics and baseline characteristics for the LION (OAB population; A), MONKEY (NDO population; B), and MARMOSET (NDO population; C) trials

**(A)**		
	Children (5–<12 y) (N = 73)	Adolescents (12–<18 y) (N = 22)
Sex, n (%)		
Male	29 (39.7)	5 (22.7)
Female	44 (60.3)	17 (77.3)
Age in years, mean (SD)	7.6 (1.6)	14.2 (1.8)
Race, n (%)		
White	62 (84.9)	16 (72.7)
Black/African American	2 (2.7)	2 (9.1)
Asian	5 (6.8)	4 (18.2)
American Indian/Alaskan Native	4 (5.5)	0
Weight in kg, mean (SD)	29.32 (8.65)	55.70 (14.42)

(B)		
	Children (5–<12 y) (N = 42)	Adolescents (12–<18 y) (N = 34)
Sex, n (%)		
Male	20 (47.6)	17 (50.0)
Female	22 (52.4)	17 (50.0)
Age in years, mean (SD)	8.3 (1.9)	13.9 (1.7)
Race, n (%)		
White	22 (52.4)	23 (67.6)
Black/African American	1 (2.4)	1 (2.9)
Asian	17 (40.5)	6 (17.6)
American Indian/Alaskan Native	0	1 (2.9)
Other	2 (4.8)	3 (8.8)
Weight in kg, mean (SD)	28.13 (8.46)	50.35 (13.29)

(C)		
	Children (6 mo–<2 y) (N = 4)	Children (2–<5 y) (N = 19)
Sex, n (%)		
Male	1 (25.0)	8 (42.1)
Female	3 (75.0)	11 (57.9)
Age in months, mean (SD)	15.88 (3.05)	39.39 (9.72)
Race, n (%)		
White	2 (50.0)	10 (52.6)
Asian	2 (50.0)	9 (47.4)
Weight in kg, mean (SD)	10.10 (1.64)	13.84 (2.65)

Table 1A reprinted from Eur Urol, 71, Newgreen D, Bosman B, Hollestein‐Havelaar A, Dahler E, Besuyen R, Sawyer W, *et al*. Solifenacin in children and adolescents with overactive bladder: results of a phase 3 randomised clinical trial, 483‐90, Copyright (2017), with permission from Elsevier.^26^

Tables 1B and 1C adapted from Franco, *et al*.^30^

Abbreviations: NDO, neurogenic detrusor overactivity; OAB, overactive bladder; SD, standard deviation.

### 3.1 Doses of solifenacin

All of the patients in the three trials initially received a starting dose of PED5, except for the four patients aged 6 months–<2 years in the MARMOSET trial who received PED2.5 (Table 2). At the time PK sampling occurred, the majority of participants (119/164 patients, 72.6% of those remaining) had been up‐titrated in all three trials and were receiving PED10 once daily.

**TABLE 2:** Summary of solifenacin dosing during the LION (OAB population; A), MONKEY (NDO population; B), and MARMOSET (NDO population; C) trials

(A)			
Visit	Dose group (mg)	Children (5–<12 y) (N = 73) n (%)	Adolescents (12–<18 y) (N = 22) n (%)
Baseline	PED5	73 (100)	22 (100)
Week 3	PED5	21 (28.8)	1 (4.5)
	PED7.5	51 (69.9)	19 (86.4)
Week 6	PED5	10 (13.7)	0
	PED7.5	18 (24.7)	4 (18.2)
	PED10	42 (57.5)	14 (63.6)
Week 9	PED5	6 (8.2)	0
	PED7.5	12 (16.4)	1 (4.5)
	PED10	47 (64.4)	16 (72.7)
Week 12	PED5	6 (8.2)	0
	PED7.5	12 (16.4)	1 (4.5)
	PED10	47 (64.4)	16 (72.7)

(B)			
Visit	Dose group (mg)	Children (5–<12 y) (N = 42) n (%)	Adolescents (12–<18 y) (N = 34) n (%)
Baseline	PED5	42 (100)	34 (100)
Week 3	PED5	8 (19.0)	8 (23.5)
	PED7.5	27 (64.3)	23 (67.6)
Week 6	PED2.5	0	1 (2.9)
	PED5	6 (14.3)	4 (11.8)
	PED7.5	5 (11.9)	7 (20.6)
	PED10	22 (52.4)	18 (52.9)
Week 9	PED2.5	0	1 (2.9)
	PED5	5 (11.9)	4 (11.8)
	PED7.5	5 (11.9)	4 (11.8)
	PED10	23 (54.8)	21 (61.8)
Week 12	PED2.5	0	1 (2.9)
	PED5	3 (7.1)	2 (5.9)
	PED7.5	6 (14.3)	5 (14.7)
	PED10	23 (54.8)	21 (61.8)
Week 24	PED2.5	0	1 (2.9)
	PED5	3 (7.1)	2 (5.9)
	PED7.5	6 (14.3)	5 (14.7)
	PED10	23 (54.8)	19 (55.9)
Week 52	PED2.5	0	1 (2.9)
	PED5	3 (7.1)	2 (5.9)
	PED7.5	6 (14.3)	5 (14.7)
	PED10	22 (52.4)	19 (55.9)

(C)			
Visit	Dose group (mg)	Children (6 mo–<2 y) (N = 4) n (%)	Children (2–<5 y) (N = 19) n (%)
Baseline	PED2.5	4 (100)	0
	PED5	0	19 (100)
Week 3	PED5	4 (100)	4 (21.1)
	PED7.5	0	14 (73.7)
Week 6	PED5	1 (25.0)	3 (15.8)
	PED7.5	3 (75.0)	7 (36.8)
	PED10	0	8 (42.1)
Week 9	PED5	0	1 (5.3)
	PED7.5	1 (25.0)	6 (31.6)
	PED10	3 (75.0)	11 (57.9)
Week 12	PED5	0	1 (5.3)
	PED7.5	1 (25.0)	6 (31.6)
	PED10	3 (75.0)	11 (57.9)
Week 24	PED5	0	1 (5.3)
	PED7.5	1 (25.0)	5 (26.3)
	PED10	2 (50.0)	12 (63.2)
Week 52	PED5	0	1 (5.3)
	PED7.5	1 (25.0)	5 (26.3)
	PED10	2 (50.0)	12 (63.2)

Abbreviations: NDO, neurogenic detrusor overactivity; OAB, overactive bladder; PED, pediatric equivalent dose.

### 3.2 PK results

**FIGURE 2:**
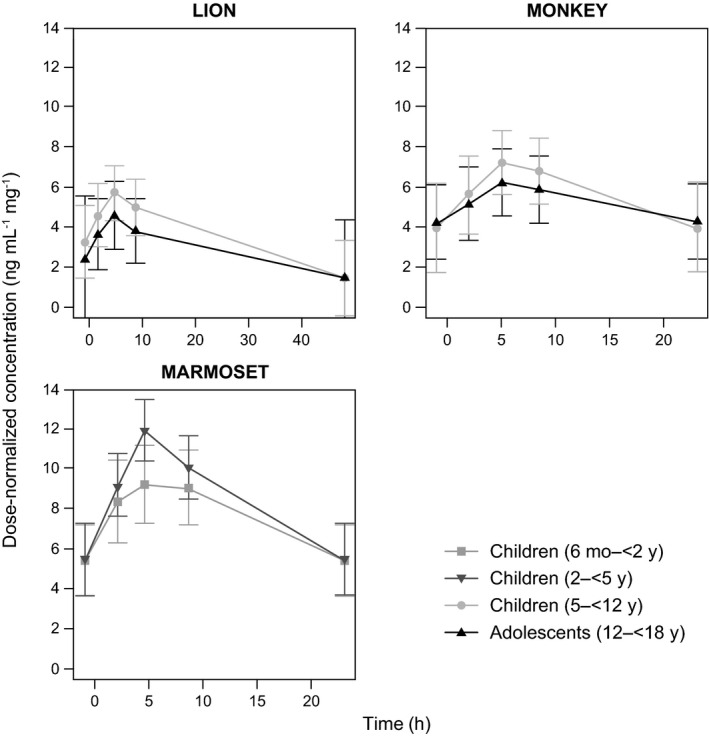
Dose‐normalized concentration‐time profiles for the patients enrolled in the LION (OAB population), MONKEY (NDO population), and MARMOSET (NDO population) trials. NDO, neurogenic detrusor overactivity; OAB, overactive bladder; SD, standard deviation. Data shown are geometric means ± SD. The curves are calculated using the nominal times for the concentration samples

The dose‐normalized concentration‐time profiles for all three trials are shown in Figure 2.

#### 3.2.1 The LION trial

The final model that was developed for the LION trial was a two‐compartment model with first‐order oral absorption and a lag time (Table 3A). The model included interindividual variability (IIV) on the apparent oral plasma clearance (CL/F), apparent central volume of distribution (V2/F), and absorption rate constant (k_a_) terms for solifenacin. Both additive and proportional residual error were included and separate error models were required for the two trials included in the dataset (the LION and GIRAFFE trials, which involved sparse and rich sampling, respectively). Fat‐free mass (FFM) was added to the clearance and volume terms as the size parameter for allometric scaling with estimated exponents, and AGP was added to CL/F and V2/F with a power model with an estimated exponent. The parameters were precisely estimated and had relatively small standard errors.

**TABLE 3:** Summary of the parameters used in the final PK model developed for the LION (OAB population; A), MONKEY (NDO population; B), and MARMOSET (NDO population; C) trials

(A)		
Parameter	Estimate	SE
CL/F (L h^–1^)	8.81^a^	0.666
AGP on CL/F^b^	–0.649	0.197
FFM on CL/F^b^	0.652	0.118
V2/F (L)	162^a^	37.2
AGP on V2/F^b^	–1.06	0.311
FFM on V2/F^b^	1.18	0.207
k_a_ (h^–1^)	0.742	0.116
ALAG (h)	0.834	0.0488
Q/F (L h^–1^)	98.1	16.7
V3/F (L)	174^c^	17.6
FFM on V3/F^b^	1.07	0.188
F1	1.12^d^	0.0905

Interindividual variability	Estimate	SE
CL/F	0.209	0.047
V2/F	0.389	0.152
k_a_	0.175	0.0643

Residual variability	Estimate	SE
Proportional	0.0174	0.0029
Additive	1.64	0.789
Proportional (single‐dose trial)	0.015	0.0044
Additive (single‐dose trial)	0 FIX	–

(B)		
Parameter	Estimate	SE
CL/F (L h^–1^)	6.22^e^	0.296
AGP on CL/F^b^	–1.18	0.164
FFM on CL/F^b^	0.431	0.109
V/F (L)	283^e^	22.6
AGP on V/F^b^	–0.184	0.265
FFM on V/F^b^	1.14	0.222
k_a_ (h^–1^)	1.38	0.33
ALAG (h)	0.934	0.0465

Interindividual variability	Estimate	SE
CL/F	0.128	0.0239
V/F	0.308	0.1321
k_a_	0.961	0.3729

Residual variability	Estimate	SE
Proportional	0.0237	0.0057
Additive	0.0674	0.4300

(C)		
Parameter	Estimate	SE
CL/F (L h^–1^)	4.03^f^	0.312
AGP on CL/F^b^	–0.382	0.183
FFM on CL/F^b^	0.933	0.246
V/F (L)	106^f^	10.3
AGP on V/F^b^	–0.337	0.357
FFM on V/F^b^	0.957	0.239
k_a_ (h^–1^)	1.22	0.421
ALAG (h)	0.688	0.172

Interindividual variability	Estimate	SE
CL/F	0.118	0.0471
V/F	0.122	0.0771
k_a_	0.878	0.412

Residual variability	Estimate	SE
Proportional	0.0122	0.0044

Abbreviations: AGP, α_1_‐acid glycoprotein; ALAG, absorption lag time; CL/F, apparent oral plasma clearance; CYP, cytochrome P450; F1, relative bioavailability; FFM, fat‐free mass; k_a_, absorption rate constant; NDO, neurogenic detrusor overactivity; OAB, overactive bladder; PK, pharmacokinetic; Q/F, apparent intercompartmental clearance; SE, standard error; V/F, apparent volume of distribution; V2/F, apparent central volume of distribution; V3/F, apparent peripheral volume of distribution.

^a^ Typical value for a patient with FFM = 24 kg and AGP = 67 ng mL^–1^.

^b^ Exponent for the power model.

^c^ Typical value for a patient with FFM = 24 kg.

^d^ Bioavailability of formulation B (single‐dose trial) relative to formulation A (single‐dose trial).

^e^ Typical value for a patient with FFM = 30 kg and AGP = 72 ng mL^–1^.

^f^ Typical value for a patient with FFM = 11.8 kg and AGP = 70 ng mL^–1^ and complete maturation of CYP3A4.

**FIGURE 3:**
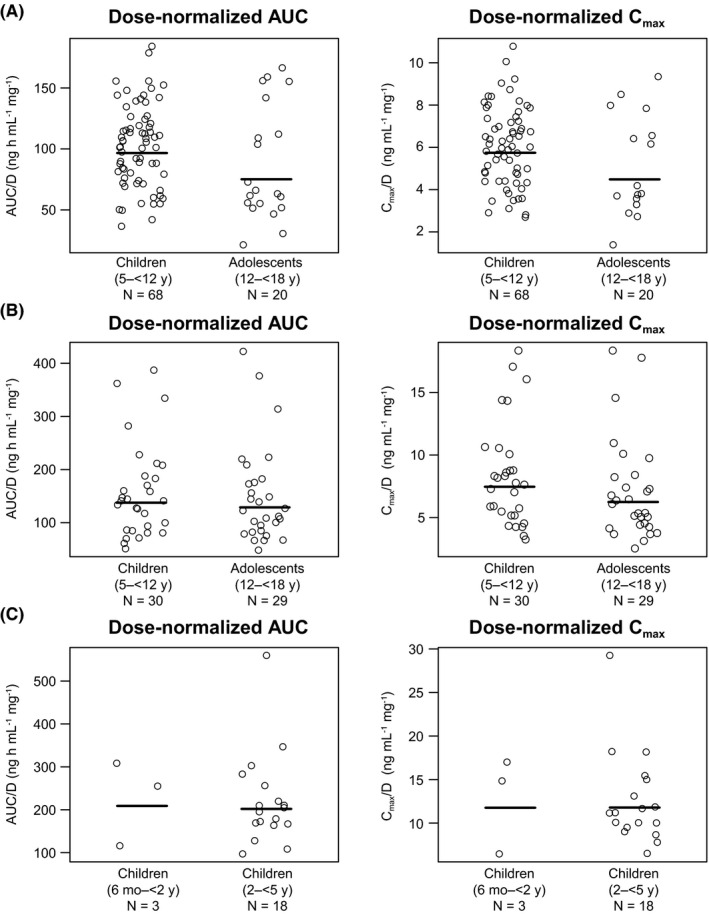
Dose‐normalized exposures according to age group for the patients enrolled in the LION (OAB population; (A), MONKEY (NDO population; (B), and MARMOSET (NDO population; (C) trials. AUC, area under the plasma concentration‐time curve; AUC/D, dose‐normalized area under the plasma concentration‐time curve; C_max_, maximum plasma concentration; C_max_/D, dose‐normalized maximum plasma concentration; NDO, neurogenic detrusor overactivity; OAB, overactive bladder. The circles represent the individual values for each patient. The line within each plot represents the geometric mean

The PK results obtained using the final model showed that mean CL/F, as well as the derived apparent volume of distribution during the terminal phase (V_z_/F), were higher in adolescents than in children (Table 4A). Median time to maximum plasma concentration (t_max_) values were very similar across both age groups and mean terminal half‐life (t_1/2_) values of 27 and 37 hours were estimated for the children and adolescents, respectively. The dose‐normalized exposure results obtained in the LION trial are shown in Figure 3A. This analysis showed that similar mean area under the plasma concentration‐time curve (AUC) and maximum plasma concentration (C_max_) results were observed for children and adolescents.

**TABLE 4:** Summary of the solifenacin PK parameters and dose‐normalized exposure metrics for the LION (OAB population; A), MONKEY (NDO population; B), and MARMOSET (NDO population; C) trials

(A)			
Parameter, geometric mean (CV%^a^)	Children (5–<12 y) (N = 66)	Adolescents (12–<18 y) (N = 18)	All patients (5–<18 y) (N = 84)
AUC/D (ng h mL^–1^ mg^–1^)	96.70 (37.11)	75.82 (58.08)	91.79 (43.14)
C_max_/D (ng mL^–1^ mg^–1^)	5.744 (33.62)	4.485 (54.28)	5.453 (39.69)
t_max_ ^b^ (h)	3.0 (2.0‐4.3)	2.8 (2.2‐3.6)	2.9 (2.0‐4.3)
t_1/2_ (h)	26.75 (26.45)	37.41 (40.56)	28.75 (32.99)
CL/F (L h^–1^)	7.797 (37.11)	9.944 (58.08)	8.214 (43.14)
V_z_/F (L)	300.9 (28.66)	536.7 (29.69)	340.7 (38.19)
C_trough_/D (ng mL^–1^ mg^–1^)	3.184 (46.05)	2.726 (68.95)	3.082 (51.26)

(B)			
Parameter, geometric mean (CV%^a^)	Children (5–<12 y) (N = 30)	Adolescents (12–<18 y) (N = 29)	All patients (5–<18 y) (N = 59)
AUC/D (ng h mL^–1^ mg^–1^)	136.8 (56.81)	128.1 (57.75)	132.5 (56.83)
C_max_/D (ng mL^–1^ mg^–1^)	7.431 (50.03)	6.210 (52.91)	6.803 (51.96)
t_max_ ^b^ (h)	3.0 (2.0‐6.0)	3.5 (2.0‐5.0)	3.0 (2.0‐6.0)
t_1/2_ (h)	23.62 (67.33)	37.96 (42.49)	29.83 (61.89)
CL/F (L h^–1^)	5.510 (56.81)	5.885 (57.75)	5.691 (56.83)
V_z_/F (L)	187.8 (61.86)	322.3 (44.45)	244.9 (61.77)
C_trough_/D (ng mL^–1^ mg^–1^)	3.810 (82.19)	4.208 (64.94)	4.001 (73.34)

(C)			
Parameter, geometric mean (CV%^a^)	Children (6 mo–<2 y) (N = 3)	Children (2–<5 y) (N = 18)	All patients (6 mo–<5 y) (N = 21)
AUC/D (ng h mL^–1^ mg^–1^)	208.9 (55.55)	202.0 (43.64)	203.0 (43.76)
C_max_/D (ng mL^–1^ mg^–1^)	11.76 (56.27)	11.78 (36.87)	11.78 (38.14)
t_max_ ^b^ (h)	4.0 (2.5‐5.0)	3.0 (2.0‐6.0)	3.0 (2.0‐6.0)
t_1/2_ (h)	17.83 (29.67)	18.05 (33.76)	18.02 (32.46)
CL/F (L h^–1^)	3.609 (55.55)	3.732 (43.64)	3.714 (43.76)
V_z_/F (L)	92.86 (63.00)	97.16 (38.09)	96.54 (40.06)
C_trough_/D (ng mL^–1^ mg^–1^)	5.677 (56.55)	5.348 (57.89)	5.394 (56.13)

Abbreviations: AUC/D, dose‐normalized area under the plasma concentration‐time curve; CL/F, apparent oral plasma clearance; C_max_/D, dose‐normalized maximum plasma concentration; C_trough_/D, dose‐normalized trough plasma concentration; CV, coefficient of variation; NDO, neurogenic detrusor overactivity; OAB, overactive bladder; PK, pharmacokinetic; t_1/2_, terminal half‐life; t_max_, time to maximum plasma concentration; V_z_/F, apparent volume of distribution during the terminal phase.

^a^ Geometric mean = exp(mean(log(x))), geometric CV% = sqrt(exp(sd(log(x))^2)–1)*100.

^b^ t_max_ is summarized in terms of median (range).

#### 3.2.2 The MONKEY trial

For the MONKEY trial, the final model was a one‐compartment model with first‐order oral absorption and a lag time (Table 3B). IIV on CL/F, apparent volume of distribution (V/F), and k_a_ as well as additive and proportional residual error were incorporated into the model. FFM was added to the clearance and volume terms as the size parameter for allometric scaling with estimated exponents, and AGP was added to CL/F and V/F with a power model with an estimated exponent. The parameters were precisely estimated and had relatively small standard errors.

In the MONKEY trial, higher mean CL/F and V_z_/F values were obtained for adolescents compared with children (Table 4B), and consistent median t_max_ values were observed across both age groups. Furthermore, mean t_1/2_ was also higher for adolescents (38 hours) in comparison with children (24 hours). The dose‐normalized mean AUC and C_max_ results were similar in both children and adolescents (Figure 3B).

#### 3.2.3 The MARMOSET trial

The final model for the MARMOSET trial was a one‐compartment model with first‐order oral absorption and a lag time (Table 3C). The model contained IIV on CL/F, V/F, and k_a_ as well as proportional residual error only. FFM was added to the clearance and volume terms as the size parameter for allometric scaling with estimated exponents, and AGP was added to CL/F and V/F with a power model with an estimated exponent. As some of the patients were < 2 years old, an ontogeny function for the CYP3A4‐mediated portion of the clearance was also included.^40^ The parameters were precisely estimated with relatively small standard errors.

Similar mean CL/F and V_z_/F values were obtained using the final model for both the children aged 6 months–<2 years and those aged 2–<5 years (Table 4C). Furthermore, consistency across the median t_max_ and mean t_1/2_ values was also observed for both age groups. The dose‐normalized mean AUC and C_max_ results were similar for both the younger and older children (Figure 3C).

## 4 DISCUSSION

It is imperative to conduct PK analyses and modeling in pediatric patients in order to describe the PK in the pediatric population, to understand the sources of variability in PK, and to inform dosing recommendations^41^ for subsequent clinical studies. This comprehensive investigation was conducted to analyze the PK of solifenacin in pediatric patients with either OAB or NDO; two populations who are physiologically distinct, but receive similar therapies.

Although solifenacin has previously shown bi‐exponential kinetics following rich sampling, the sparse sampling in the MONKEY and MARMOSET trials did not allow a full characterization of the profile shape, and therefore the final structural model for patients with NDO was a one‐compartment model. By pooling the data from the LION trial with the richly‐sampled, single‐dose GIRAFFE trial, it was possible to characterize the full solifenacin profile and achieve a two‐compartment model for patients with OAB. All models included first‐order oral absorption and a lag time, and IIV on CL/F, V2/F or V/F, and k_a_. Proportional residual error was included for all of the models; the models for the LION and MONKEY trials also contained additive residual error.

Two covariates, FFM and AGP, were added to the clearance and volume terms for all of the models. FFM includes muscle, bone, vital organs, and extracellular fluid and can be used to explain variability in drug clearance.^42^, ^43^ Other size metrics such as weight and lean body mass had been investigated in previous solifenacin population PK models, with FFM found to be the most robust factor. In addition, as FFM was used as a metric in clinical decisions, such as the construction of the pediatric dosing tables used in clinical studies, this covariate was selected a priori for addition to the base model. Including AGP on the clearance and volume terms is also reasonable from a physiological perspective. For drugs like solifenacin with low intrinsic clearance and high plasma protein binding to AGP,^34^, ^44^ clearance is approximately proportional to the fraction unbound. As a plasma protein, AGP would also have an impact on the distribution of solifenacin out of the plasma. An ontogeny function for the CYP3A4‐mediated portion of solifenacin clearance was also included in the model for the MARMOSET trial; this step was taken as some of the patients in this investigation were < 2 years old and therefore full maturation of their CYP3A4 activity may not have occurred.^40^

This investigation showed that the PK characteristics of solifenacin were similar in pediatric patients with OAB and NDO. This finding is particularly reassuring given the differences in physiological characteristics between the two populations, for example the decreased muscle mass that is apparent in children with neurogenic bladder.^45^ Overall analysis showed that apparent clearance was similar across the trials (after adjusting for FFM and AGP and including the assumption that CYP3A4 was fully mature). Analysis of the PK parameters across age groups indicated that mean CL/F and V_z_/F were higher in adolescents than in children, due to adolescents having a larger FFM. Median t_max_ was similar across both the older and younger age groups across all three trials and varied between 2.8 and 4.0 hours. The mean t_1/2_ results were higher in adolescents compared with children, although variation was observed in the data obtained. Similar dose‐normalized results were obtained for the younger and older pediatric patients with OAB or NDO. This finding was expected given that weight‐based dosing regimens were employed.

The target AUC for pediatric patients in these trials was 889 ng h mL^–1^ (5th–95th percentile: 421‐1896 ng h mL^–1^) for PED10; this range was derived from a study in healthy adults who received 10 mg of the same solifenacin suspension formulation (Study 905‐CL‐080; unpublished data on file, Astellas Pharma). In total, 74%, 85%, and 90% of the pediatric patients from the LION, MONKEY, and MARMOSET trials, respectively, had exposure results that fell within the percentile range from the adult study. This finding demonstrates the utility of the dose titration weight‐based regimens for solifenacin that were used in these pediatric trials and the models that were developed to characterize the PK results.

The PK findings from a single‐dose trial in pediatric patients (5–<18 years) with NDO have been presented previously (ELEFANT; Study 905‐CL‐079).^28^ Although slightly higher mean CL/F, t_1/2_, V_z_/F, and median t_max_ results were observed in this single‐dose trial, the PK results obtained were broadly similar to the findings from the MONKEY trial. Furthermore, slightly lower mean AUC and C_max_ results were typically obtained in the LION trial compared with the single‐ascending dose PK trial in pediatric patients with OAB (GIRAFFE), although similar overall findings were observed.^38^ In addition, the results of ELEFANT and GIRAFFE indicated that similar PK data were observed following the administration of single doses of solifenacin to patients with OAB or NDO.

In conclusion, the PK of solifenacin in pediatric OAB or NDO populations were characterized through population PK modeling. Although higher mean CL/F, V_z_/F, and t_1/2_ were observed for adolescents than for children, similar median t_max_ results were observed for both age groups. Dose‐normalized exposure results were similar for both the younger and older patients with either condition. Overall, this investigation showed that pediatric patients with OAB or NDO demonstrate similar PK characteristics for solifenacin.

## AUTHOR CONTRIBUTORS

All the authors have drafted the manuscript and revised the manuscript critically for important intellectual content, have provided final approval of the manuscript version to be published, and agreed to be accountable for all aspects of the manuscript. ST had full access to all the data included in the manuscript and takes responsibility for the integrity of the data and the accuracy of the data analysis; MdA, WK, AH‐H, FV, and DN were involved in the initial trial designs; MdA, AH‐H, FV, and DN were involved in any subsequent amendments to the trial designs; AH‐H, FV, and DN were involved in the trial implementation; AH‐H and FV were involved in the enrollment of the patients; ST and WK performed the pharmacokinetic analysis; JM was responsible for the solifenacin plasma bioanalysis; ST, MdA, WK, and DN were involved in the data interpretation; ST, MdA, AH‐H, and DN were involved in writing the trial reports; and all the authors were involved in reviewing the trial data and editing the final clinical trial report.

## DATA SHARING STATEMENTS

Researchers may request access to anonymized participant‐level data, trial‐level data, and protocols from Astellas sponsored clinical trials at www.clinicalstudydatarequest.com. For the Astellas criteria on data sharing see: https://clinicalstudydatarequest.com/Study‐Sponsors/Study‐Sponsors‐Astellas.aspx.

## DISCLOSURES

All the authors are current or former employees of either Astellas Pharma Europe B.V. or Astellas Pharma Global Development Inc. The authors thank the LION, MONKEY, and MARMOSET trial investigators, and all the patients and their parents/legal representatives who took part in the trials. These trials were funded by Astellas Pharma Europe B.V. Medical writing support was provided by Michael Parsons, PhD, CMPP of Elevate Scientific Solutions, who provided permission to be named in this section, and funded by Astellas Pharma Global Development, Inc. The authors also thank the contract research organization involved in conducting these trials, PPD Global Limited, Cambridge, UK.

## Supporting information

Table S1
