# Supplementary material for: Pharmacokinetics of solifenacin in pediatric populations with overactive bladder or neurogenic detrusor overactivity
Source: Pharmacol Res Perspect. 2020 Nov 24;8(6):e00684. doi: 10.1002/prp2.684 (PMC7685239; doi:10.1002/prp2.684)
Supplement: Supplementary file 1 — Table S1 [file PRP2-8-e00684-s001.docx]

Table S1 Inclusion and exclusion criteria for the LION (OAB population; A), MONKEY (NDO population; B), and MARMOSET (NDO population; C) trials

A

| **Inclusion criteria** | **Exclusion criteria** |
| --- | --- |
| **At screening** |  |
| OAB (symptoms of urgency) according to ICCS criteria | Current constipation as evaluated by the Rome III criteria |
| Male or female, 5–<12 or 12–<18 years of age | Suffered from chronic UTI or had > 3 UTIs 2 months prior to screening |
| Weight and height were within normal percentiles for their age (3rd–97th percentile) | Any catheterization within 2 weeks prior to screening |
| Patient and patient’s parent(s)/legal guardian(s) were able to comply with the trial requirements and the concomitant medication restrictions | History or presence of any malignancy within 5 years prior to screening |
| Written informed consent had to be obtained from the patient’s parent(s)/legal guardian(s) prior to any trial-related procedures; assent (patient) where appropriate was given | Participated in another clinical trial and/or had taken an investigational product within 30 days (or five half-lives of the drug, or the limit set by national law, whichever was longer) prior to screening |
| Female patients of childbearing potential and sexually active agreed to use a reliable form of birth control for the duration of the trial and for ≥ 1 month afterwards. Sexually active male patients agreed to use a condom for the duration of the trial and for ≥ 1 month afterwards | Electrostimulation or bladder training within 2 weeks prior to screening |
| **At baseline** |  |
| Fulfil all screening inclusion criteria | Fulfil none of the screening exclusion criteria |
| Daytime incontinence with ≥ 4 episodes of incontinence during a 7-day period of baseline assessment (confirmed by diary) | Maximum voided volume > EBC for age ([age +1] x 30) in mL or a maximum voided volume > 390 mL |
|  | PVR volume > 20 mL |
|  | UTI confirmed by urinalysis (urine culture containing > 100 000 cfu mL^–1^) |
| **At screening, start of run-in period, and at baseline** | |
|  | Daily voiding frequency < 5 |
|  | Extraordinary daytime urinary frequency according to the ICCS definition. All three criteria had to apply   - Daytime voiding frequency was at least once hourly - Mean voided volumes were < 50% of EBC - Nocturnal bladder behavior was normal for the age of the patient |
|  | Uroflow indicative of non-OAB pathology |
|  | Monosymptomatic enuresis |
|  | Polyuria (> 75 mL kg^–1^ body weight^–1^ 24 hours^–1^) |
|  | Central or nephrogenic diabetes insipidus |
|  | Dysfunctional voiding |
|  | Congenital anomalies affecting LUT function |
|  | Hematuria ≥ ++ on dipstick test |
|  | Kidney/bladder stones or other persistent local pathology that could cause urinary symptoms |
|  | One of the following gastrointestinal problems: partial or complete bowel obstruction, decreased motility, or at risk of gastric retention |
|  | QTcB > 440 ms, a history of QTc prolongation, or at risk of QT prolongation |
|  | Any relevant history or presence of malignancy related to the urogenital tract |
|  | Clinically significant unstable medical condition or disorder, which, in the opinion of the investigator, precluded the patient |
|  | Serum creatinine ≥ 2 times the ULN |
|  | AST or ALT ≥ 2 times the ULN, or bilirubin ≥ 1.5 times the ULN |
|  | Breastfeeding, pregnant, or intended to become pregnant |
|  | Known or suspected hypersensitivity to solifenacin (or other anticholinergics), or any of the excipients used |
|  | Not likely to complete the trial for any reason in the opinion of the investigator |
|  | Parent(s)/legal guardian(s) of the patient were employees of the Astellas Group, third parties associated with the trial, or the clinical trial site team executing the trial |
|  | Planned electrostimulation or bladder training (outside of trial-related urotherapy) at any time during the trial |
|  | Using or had used prohibited prior and/or concomitant medication(s) |
|  | Using restricted medication(s) |
|  | History of glaucoma |

B

| **Inclusion criteria** | **Exclusion criteria** |
| --- | --- |
| **At screening** |  |
| Male or female, 5–<18 years of age | Breastfeeding, pregnant, or intended to become pregnant |
| Documented diagnosis of NDO | Known genitourinary condition (other than NDO) that might have caused incontinence |
| Patient and patient’s parent(s)/legal guardian(s) were able to comply with the trial requirements and the concomitant medication restrictions | Undergone bladder augmentation surgery |
| Practicing CIC | Bladder capacity < 25% of expected age-related capacity |
| Adjudged suitable for a regimen of four to six CICs per day for the duration of the trial | Electrostimulation within 2 weeks prior to screening and at any time during the trial |
| Treated with an antimuscarinic drug for ≥ 6 months | Vesicoureteral reflux grade 3-5 |
| Weight was within normal percentiles for their age | Kidney/bladder stones or other pathology causing urinary symptoms |
| Bowel dysfunction had to be actively managed in sufferers | Indwelling urinary catheter within 4 weeks prior to screening |
| Able to swallow the trial medication | One of the following gastrointestinal problems: partial or complete bowel obstruction, decreased motility, or at risk of gastric retention |
| Female patients of childbearing potential and sexually active agreed to use a reliable form of birth control for the duration of the trial and for ≥ 1 month afterwards. Sexually active male patients agreed to use a condom for the duration of the trial and for ≥ 1 month afterwards | Existing fecal impaction |
| Written informed consent had to be obtained from the patient’s parent(s)/legal guardian(s) prior to any trial-related procedures; assent (patient) where appropriate was given | QTcB > 440 ms, a history of QTc prolongation, or at risk of QT prolongation |
| Agreed not to participate in another interventional study while on treatment | History or presence of any malignancy within 5 years prior to screening; any relevant history or presence of malignancy related to the urogenital tract |
|  | Clinically significant or unstable medical condition or disorder, which, in the opinion of the investigator, precluded the patient |
|  | Central or congenital nephrogenic diabetes insipidus |
|  | Severe renal impairment (GFR < 30 mL min^–1^) |
|  | AST or ALT ≥ 2 times the ULN, or total bilirubin ≥ 1.5 times the ULN |
|  | Any other clinically significant out-of-range urinalysis, biochemistry, or hematology results |
|  | Known or suspected hypersensitivity to solifenacin (or other anticholinergics), any of the excipients used, or previous severe hypersensitivity to any drug |
|  | Participated in another clinical trial and/or had taken an investigational product within 30 days (or five half-lives of the drug, or the limit set by national law, whichever was longer) prior to screening |
|  | Used prohibited medications and restricted medications |
|  | Received intravesical botulinum toxin within 9 months prior to screening |
|  | Parent(s)/legal guardian(s) of the patient were employees of the Astellas Group, the CRO involved, or the investigator site that executed the trial |
|  | History of glaucoma |
| **At baseline** |  |
| Diagnosis of NDO had to be confirmed by urodynamics demonstrating the presence of involuntary detrusor contractions involving a detrusor pressure increase > 15 cmH_2_O above baseline | UTI confirmed by urinalysis (urine culture containing > 100 000 cfu mL^–1^) |
|  | Recurrent UTI between screening and baseline |
|  | DSD or surgically corrected underactive urethral sphincter and did not meet the urodynamic inclusion criteria for NDO |

C

| **Inclusion criteria** | **Exclusion criteria** |
| --- | --- |
| **At screening** |  |
| Male or female, 6 months–<5 years of age |  |
| Minimum weight of 6 kg |  |
| Previous myelomeningocele |  |
| Documented diagnosis of NDO; which had to be confirmed by urodynamic assessments at baseline |  |
| Suffered from DSD |  |
| Practicing CIC |  |
| Adjudged suitable for a regimen of four to six CICs per day for the duration of the trial |  |
| Able to swallow the trial drug |  |
| Patient’s parent(s)/legal guardian(s) were able to comply with the trial requirements and the concomitant medication restrictions |  |
| Written informed consent had to be obtained from the patient’s parent(s)/legal guardian(s) prior to any trial-related procedures |  |
| Patient’s parent(s)/legal guardian(s) agreed not to allow patient to participate in another interventional study while on treatment and throughout the pretreatment period |  |
| **At screening or baseline** |  |
|  | Bladder capacity < 25% of expected age-related capacity |
|  | Vesicoureteral reflux grade 3-5 |
|  | Known genitourinary condition (other than NDO) that might have caused incontinence |
|  | Indwelling urinary catheter within 4 weeks prior to the trial center visit |
|  | Undergone bladder augmentation surgery |
|  | Surgically-corrected underactive sphincter |
|  | Electrostimulation within 2 weeks prior to the trial center visit |
|  | Received intravesical botulinum toxin within 9 months prior to screening |
|  | UTI confirmed by urinalysis (urine culture containing > 100 000 cfu mL^–1^) at baseline |
|  | Used prohibited medications |
|  | Kidney/bladder stones or other pathology causing urinary symptoms |
|  | Central or congenital nephrogenic diabetes insipidus |
|  | Bowel dysfunction, unless the condition was being actively managed |
|  | Fecal impaction |
|  | Severe gastrointestinal condition or any of the following gastrointestinal problems: partial or complete bowel obstruction, decreased motility, or at risk for gastric retention |
|  | History of glaucoma |
|  | Known or suspected hypersensitivity to solifenacin, any of the excipients used, or previous severe hypersensitivity to any drug |
|  | Patient was suffering from malnutrition or was severely overweight |
|  | QTcB > 440 ms, a history of QTc prolongation, or at risk of QT prolongation |
|  | Severe renal impairment (GFR < 30 mL min^–1^) |
|  | AST or ALT ≥ 2 times the ULN, or total bilirubin ≥ 1.5 times the ULN |
|  | Any other clinically significant out-of-range urinalysis, biochemistry, or hematology results |
|  | Current or previous history of epilepsy |
|  | History or presence of any malignancy |
|  | Clinically significant or unstable medical condition or disorder, which, in the opinion of the investigator, precluded the patient |
|  | Participated in another clinical trial and/or had taken an investigational drug within 30 days (or five half-lives of the drug, or the limit set by national law, whichever was longer) prior to the trial center visit |
|  | Parent(s)/legal guardian(s) of the patient were employees of the Astellas Group, the CRO involved, or the investigator site that executed the trial |
|  | Breast-fed by a woman taking any prohibited medication or fed with a milk product in which the presence of prohibited medication ingredients could not be excluded |

Table S1A reprinted from Eur Urol, 71, Newgreen D, Bosman B, Hollestein-Havelaar A, Dahler E, Besuyen R, Sawyer W, *et al*. Solifenacin in children and adolescents with overactive bladder: results of a phase 3 randomised clinical trial, 483-90, Copyright (2017), with permission from Elsevier.[^1^](#_ENREF_1)

Tables S1B and S1C adapted from Franco, et al.[^2^](#_ENREF_2)

Abbreviations: ALT, alanine aminotransferase; AST, aspartate aminotransferase; CIC, clean intermittent catheterization; CRO, contract research organization; DSD, detrusor sphincter dyssynergia; EBC, expected bladder capacity; GFR, glomerular filtration rate; ICCS, International Children’s Continence Society; LUT, lower urinary tract; NDO, neurogenic detrusor overactivity; OAB, overactive bladder; PVR, post-void residual; QT, QT interval; QTc, QT interval corrected for heart rate; QTcB, QT interval corrected for heart rate by Bazett’s formula; ULN, upper limit of normal; UTI, urinary tract infection.

## References

1. Newgreen D, Bosman B, Hollestein-Havelaar A, et al. Solifenacin in children and adolescents with overactive bladder: results of a phase 3 randomised clinical trial. *Eur Urol*. 2017;71:483-490.

2. Franco I, Hoebeke P, Baka-Ostrowska M, et al. Long-term efficacy and safety of solifenacin in pediatric patients aged 6 months to 18 years with neurogenic detrusor overactivity: results from two phase 3 prospective open-label studies. *J Pediatr Urol*. 2020;16:180.e1-180.e8.
